# Development of a composite index for the assessment of food systems in the Philippines

**DOI:** 10.3389/fnut.2025.1566014

**Published:** 2025-04-30

**Authors:** Maria Julia Golloso-Gubat, Angelina Felix, Nancy A. Tandang, Cecilia Cristina S. Acuin, Prudenciano U. Gordoncillo

**Affiliations:** ^1^Department of Science and Technology, Food and Nutrition Research Institute, Taguig, Philippines; ^2^Institute of Human Nutrition and Food, University of the Philippines, Los Baños, Philippines; ^3^Institute of Statistics, University of the Philippines, Los Baños, Philippines; ^4^Department of Agricultural and Applied Economics, University of the Philippines, Los Baños, Philippines

**Keywords:** food system, composite index, metrics, indicators, sub-national assessment, Philippines

## Abstract

Food system assessment is vital in providing informed decisions for relevant transformations and policy shifts. In the present study, we developed and a composite index that can be utilized to quantitatively assess the status and/or performance of the food systems in the Philippines. Initially, a set of indicators were generated by Delphi approach, and relevant local data were used to develop algorithms to quantitatively operationalize the indicators which were subsequently grouped into domains by Principal Component Analysis (PCA). Equal weights were applied to indicators, and the linear additive aggregation technique was employed. The robustness of the model was also tested by uncertainty and sensitivity tests. Finally, the utility of the index was tested to describe the status of food systems in the Philippines at the across regions. Results indicate differences in regional food system scores; Central Luzon, CALABARZON, and CAR have higher scores than the other regions, while Bicol, Western Visayas, and Davao obtained relatively low scores. The sub-national level assessment indicates differences in food system concerns and priority areas across the country, providing implications for context-specific program and policy development.

## 1 Introduction

The application of the food system approach is deemed particularly relevant in low–and middle-income countries (LMICs) where food systems are fast changing, and transformation opportunities may be valuable for economic development (1). By these transformations, food systems must be able to deliver food and nutrition security and contribute to building equitable livelihoods and sustainable communities. To facilitate and guide these transformations however, an assessment of the current food system status and/or performance is an essential prerequisite. Food system metrics have been developed with the collective goal of informing food system stakeholders in developing relevant policies and/or programs. Archarya, et al. (2) proposed a set of metrics for assessing sustainable nutrition security, while the methods developed by Prosperi, et al. (3), largely considers vulnerability indicators for the assessment of food systems in the Mediterranean region. Sustainability considerations were integrated in the multi-indicator food system metrics developed by a group of stakeholders convened by the ILSI Research Foundation (4). The SUSFANS metrics on the other hand was developed to assess food and nutrition security in the context of European food systems (5). Similarly, the Food and Agriculture Organization’s (FAO) compendium of indicators for nutrition-sensitive agriculture offers a set of recommended indicators for measuring identified outcomes of nutrition-sensitive investments in the nutrition-agriculture pathway within the premise of the European food systems (6). Recently, a food system metrics was developed consisting of indicators based on data availability in selected LMICs, i.e., Ethiopia, Nigeria, Vietnam, and Bangladesh (7).

The internationally available food system metrics are contextualized using different methodological frameworks, and most of the indicators have been field-tested especially in developed countries (8). Also, the indicators for these metrics are largely contextualized at the national scale and data for food system assessment at smaller geographic scales are largely limited. While there is a recent effort to harmonize food system indicators, the authors acknowledge that its utility is limited to specific island countries and island states (8). In the Philippines, the system of governance and resource management is highly devolutionized at the regional, provincial, and/or municipal levels. Thus, food system assessment is more meaningful (and transformations more relevant) if it is established and operationalized in this framework, utilizing locally available pertinent data.

The present study developed a composite index for sub-national level food system assessment, and tested its utility across the seventeen (17) regions in the Philippines.

## 2 Materials and methods

This study involved five (5) key steps: (i) identification of indicators; (ii) development of algorithms; (iii) construction of the index; (iv) test of robustness; and, (v) adaptation of the index to assess the status of food systems in the Philippine regions.

### 2.1 Identification of indicators

**Figure 1:**
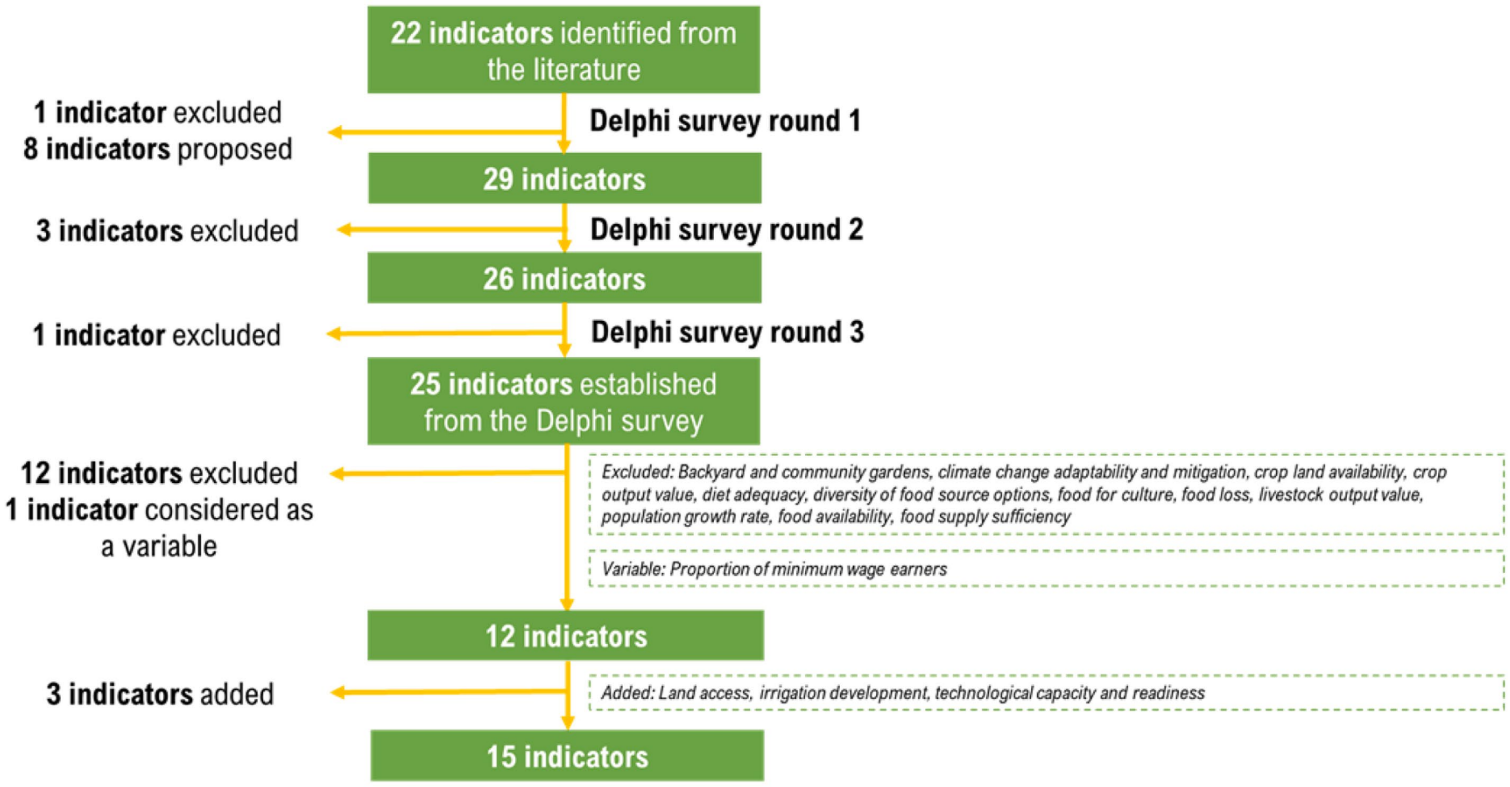
Flowchart for indicator retention.

Indicators were determined by conducting a review of the literature, and an iterative feedback process (Delphi survey) involving stakeholders from various fields of discipline relevant to the local food system. From a comprehensive review of internationally and locally accessible literature, an initial set of twenty-two (22) indicators with corresponding definitions relevant to the Philippine context, was developed. The three-round Delphi survey was conducted using a web-based survey platform. In the first round, questions focused on the proposed set of indicators with definitions and sought alternatives suggested by the members of the Delphi panel. The second round of the Delphi survey consisted of feedback from the results in the first round of the survey and a set of questions soliciting consensus on the propositions and alternatives indicated by the Delphi panel members in round 1. The last round also included feedback from the previous round, and questions soliciting consensus on the proposed definition of selected indicators and alternatives that were proposed in round 2. For all three (3) rounds, a medium threshold consensus level (=70% agreement) (9) was considered as the acceptable consensus level for an indicator to be considered in the next round of the survey. The Delphi panel of experts (*n* = 19) consisted of 12 females (63%) and seven males (37%) with ages ranging from 26 to 66 years (mean age = 43 years) and different fields of expertise. Response rate was high (round 1 = 100%; round 2 = 95%; round 3 = 100%) and the representation of the different academic disciplines was relatively stable in the three rounds (Table 1). Of the twenty-five (25) identified indicators established in the Delphi survey, twelve (12) indicators were excluded due to lack of sufficient and appropriate quantitative data for its operationalization, and one (1) indicator was considered as a variable for the operationalization of another indicator. Three (3) indicators were deemed relevant and were added, thus a total of fifteen (15) indicators identified (Figure 1).

**Table 1:** Response rate in the Delphi process, by subject matter expertise.

Discipline	*n*	No. of responses obtained		
		Round 1	Round 2	Round 3
Agriculture	2	2	1	2
Agricultural economics	2	2	2	2
Food industry	2	2	2	2
Human nutrition	2	2	2	2
Public health	2	2	2	2
Social development	2	2	2	2
Policy development	2	2	2	2
Environment	2	2	2	2
Statistics	1	2	2	2
Food supply chain and trade management	2	2	2	2
Total/Response rate	19	19 (100%)	18 (95%)	19 (100%)

### 2.2 Development of algorithms

Data from different sources were evaluated in terms of their relevance, availability and accessibility, completeness, and time. In assessing the relevance, the main consideration was the aptness of the data to suitably define and quantitatively operationalize the identified indicators. Readily available data were downloaded from the identified data sources and compiled for subsequent data processing. Otherwise, data access was requested to pertinent agencies/organizations via e-mail or by accomplishing the data request form, whichever is applicable. The most recent datasets before the pandemic (i.e., 2015–2019) with values or information for the seventeen (17) regions in the country were considered.

Algorithms were developed to quantitatively operationalize each indicator and to derive a dataset for the subsequent construction of the composite index data model. The indicators are summarized in Table 2.

**Table 2:** PCA-derived grouping of indicators into domains.

Domain	Description	Indicators
Socio-economic and socio-political conditions	This domain consists of indicators that describes the socio-economic conditions among households and assess the socio-political landscape defining the local food system, e.g., technological status, conditions of land tenure security, status of improving roads and irrigation, and the provisions of relevant policies.	Poverty incidence WASH Food expenditure status Food security status Technological capacity and readiness Road density Access to land Irrigation development Policy environment for resilience Policy environment for nutrition
Food waste and R&D support	This domain quantifies household-level food waste in local food systems. It also gauges the level of local government’s support to the advancement of R&D	Plate waste Support for technological transformation
Affordability, diversity, and sufficiency of food	This domain consists of indicators that characterize the ability of the food system to deliver food security	Food supply diversity Food affordability
Nutritional adequacy	This domain puts particular focus on the nutritional adequacy of the population’s diets	Population share with adequate energy and nutrient intakes

#### 2.2.1 Food supply diversity

In this study, food supply diversity is an indicator that characterizes the diversity of the food supply in the country utilizing region-level data on food production from the Regional Agricultural Production Accounts Report (10). By adopting the formula for Shannon Diversity of Food Supply proposed by Gustafson, et al. (4) (Equation 1), food supply diversity is operationalized as follows:


(1) $$\mathrm{Shannon}\;\mathrm{Diversity}=-\sum_{i}s_{i}\ln\left(s_{i}\right)$$

In this equation, $s_{i}$ represents the share (by weight) of the *i*_th_ food item in the food supply. The proportion or share by weight ( $s_{i}$ ) of each particular food commodity can be derived by dividing the volume of its production (expressed in metric tonnes) by the total volume of production for all food commodities. For example, the proportion of “*palay*” (unprocessed rice with husk) can be derived by dividing its volume of production over the total volume of production for all crops, livestock and poultry, and fisheries in the dataset. After obtaining the proportions, the corresponding natural logarithm ( $\ln\left(s_{i}\right)$ ) values for each food item were calculated using the LN function. Subsequently, the proportions obtained were multiplied with their corresponding natural logarithm values ( $s_{i}\ln\left(s_{i}\right)$ ) and were summed up using the SUM function.

#### 2.2.2 Population share with adequate energy and nutrient intakes

The 2015 Updating of the Nutritional Status of Filipino Children and Other Population Groups Dietary Survey (11) includes region-level data on the percentage of households with energy intake levels meeting 100% of the Recommended Energy Intakes (REI) and nutrient intakes meeting the Estimated Average Requirements (EAR) established in the Philippine Dietary Reference Intakes (PDRI). However, dietary intake data are limited to selected nutrients including protein, iron, calcium, vitamin A, vitamin C, thiamin, riboflavin, and niacin. This indicator is therefore operationalized as the average of the proportion of households in the region with intake levels meeting the requirements for energy and these nutrients.

#### 2.2.3 Food affordability

The formulation of food affordability as an indicator in this study takes into consideration the affordability of a nutritious diet based on the prevailing daily minimum wage rates in different areas of the country. To determine the average minimum daily wage rates, the prevailing minimum daily wage rates were averaged for all sectors (non-agriculture, agriculture, retail/ service, manufacturing, cottage/ handicraft, *etc.*) per region using the data obtained from PSA (12). To determine the affordability of a nutritious diet, the estimated cost of a nutritious diet (13) is divided by the calculated average minimum wage in each region. The derived score represents the portion of the cost of a nutritious diet that can be afforded by the population in a particular region, given the average minimum wage rate prevailing in the area.


(2) $$\mathrm{Food}\;\mathrm{affordabilit}y_{i}=\frac{\mathrm{CotN}D_{i}}{\min\;\mathrm{wag}e_{i}}\;x\;100\%$$

where:

$\mathrm{CotN}D_{i}$ is the cost of a nutritious diet in the $i_{th}$ region

$\min\;\mathrm{wag}e_{i}$ is the average minimum wage rate in the $i_{th}$ region

#### 2.2.4 Food expenditure status

This indicator defines the share of household income spent on food across the country. The 2018 Family Income and Expenditure Survey (FIES) (14) includes data on major expenditure groups and their respective share to the total annual family expenditure at the national level and across different regions. Data on the food expenditure were obtained. Values are expressed as percentage in 0 to 100 scale and were directly adopted to quantify this indicator.

#### 2.2.5 Poverty incidence

Poverty incidence is defined as the proportion of Filipino families with income levels that are not sufficient to meet the minimum basic food and non-food needs (15). Estimates (in percentage) of poverty incidence among families are reported for different regions in the country in the 2018 Full Year Official Poverty Statistics of the Philippines (15) and these were adopted for use in the present study to define this indicator. To operationalize this indicator, Equation 3 was adopted for use.


(3) $$100-\mathrm{Poverty}\;\mathrm{incidenc}e_{i}$$

where: $\mathrm{Poverty}\;\mathrm{incidenc}e_{i}$ is the poverty incidence in the $i_{th}$ region

#### 2.2.6 Water, sanitation, and hygiene

This study operationalizes WASH as an indicator using data from the 2019 APIS (16). The percentage of families in different regions with basic service levels for the three variables, i.e., water, sanitation, and hygiene, were averaged to derive the indicator score.

#### 2.2.7 Access to land

In this study, access to land as an indicator is considered a metric of the government’s initiatives to improve the quality of agricultural productivity and social equity in the country by enhancing rural growth and development through equitable land ownership. Data were obtained from the latest publicly available results of the Census of Agriculture and Fisheries (CAF) (17) This indicator is defined as the proportion of fully owned holding/farm parcels. This is derived by using the following equation:


(4) $$\begin{array}{l} \mathrm{Access}\;to\;land_{i}\\ =\frac{\mathrm{Number}\;\mathrm{of}\;\mathrm{fully}\;\mathrm{owned}\;\mathrm{farm}\;\mathrm{parcel}s_{i}}{\mathrm{Total}\;\mathrm{number}\;\mathrm{of}\;\mathrm{farm}\;\mathrm{parcel}s_{i}}\;x\;100\% \end{array}$$

#### 2.2.8 Irrigation development

This study adopts irrigation development as an indicator to measure the extent of government support for agriculture. The indicator is expressed as the ratio of the irrigated area collectively developed by the different agencies and private sectors vs. the estimated irrigable area per region (18). Values are expressed in percentages and were directly adopted from the data source.

#### 2.2.9 Support to technological development

Data from the DOST‘s R&D statistics for technical achievement and competitiveness were utilized to operationalize this indicator. The 2018 R&D intensity, expressed as the proportion of R&D expenditure to the corresponding Gross Domestic Product (GDP) of a particular geographic entity is obtained from the Compendium of S&T Statistics (19) and adopted for use in the present study.

#### 2.2.10 Road density

The Cities and Municipalities Competitiveness Index (CMCI) is used to evaluate and rank cities and municipalities in the Philippines based on their ability to be productive and improve the standards of living given their resources (20). Rankings are based on the calculated overall competitiveness scores for the four (4) indicators namely: economic dynamism, government efficiency, infrastructure, and resilience; each of which consists of several sub-indicators (20). The CMCI defines the indicator Infrastructure as the physical building blocks that connect, expand, and sustain a locality and its surroundings to enable the provision of goods and services (20). This indicator consists of 10 sub-indicators including Road Network which is adopted for use in the present study. The road network score was estimated as the proportion of the total length of roads (including bridges) to the total land area in a locality (20), and the 2021 CMCI Scores for this sub-indicator are adopted for use in the present study.

#### 2.2.11 Technological capacity and readiness

Like the previous indicator, data were accessed through the CMCI data portal.^1^ The file contained the 2019 scores for all localities arranged alphabetically. These localities were identified and re-grouped according to the region that they belong to, based on the UACS to develop region-level data, and the scores were summed to derive region-level scores.

#### 2.2.12 Food security status

Assessment of household food security status is included in the conduct of the 2015 DOST-FNRI NNS. In this survey, the use of the Household Food Insecurity Access Scale (HFIAS) is employed – an assessment tool developed through the USAID’s Food and Nutrition Technical Assistance (FANTA) Project. The 2015 NNS-Food Security Survey identified the proportion of food-secure and food-insecure households in the Philippines. These values were directly imported for use in the present study.

#### 2.2.13 Plate waste

In the Philippines, data on food waste is largely lacking, and available data is limited to household plate waste as a component of the 2015 DOST-FNRI NNS Dietary Survey. Plate waste is simply the post-consumption waste that is either discarded or fed to pets, measured through actual weighing (11). Data on household plate waste across regions were presented by food group and expressed in grams. To obtain the score for this indicator, the total amount of plate waste (in grams) across all food groups was obtained for each region. The value was then divided by the overall amount of household plate waste in the country and multiplied by a factor of 100 to estimate the region’s percentage share in the amount of household plate waste. Subsequently, the derived value was subtracted from 100 to estimate the percentage share of unwasted food in the region. The value derived from Equation 5 is subtracted from 100.


(5) $$\mathrm{Plate}\;\mathrm{wast}e_{i}=\frac{\sum \mathrm{wast}e_{i}}{\mathrm{plate}\;\mathrm{wast}e_{PH}}\;x\;100$$

#### 2.2.14 Policy environment for resilience

To operationalize this indicator, two (2) sub-indicators from the CMCI data portal (see text footnote 1) Resiliency pillar were adopted: (i) Disaster Risk Reduction Plan; and (ii) Budget for Disaster Risk Reduction and Management Plan (DRRMP). The former is defined as the “presence of emergency response/ disaster risk reduction plans in a given geographic area,” while the latter is defined as “contingency fund for disaster as % of total LGU budget” (CMCI, n.d.). In the present study, scores for these sub-indicators were averaged.

#### 2.2.15 Policy environment for nutrition

NNC (21) Governing Board issued a resolution on the use of Monitoring and Evaluation of Local Level Plan Implementation (MELLPI) Pro as a tool for monitoring and evaluation of nutrition programs and performance at the local level. While it is ideal to use region-level results from MELLPI Pro, consolidated records are not yet available and thus, this indicator is operationalized solely based on the availability of Regional Plan of Action for Nutrition (RPAN) in the region. As defined in the present study, the indicator policy environment for nutrition pertains to the “presence of nutrition policies/ action plans in a given geographic area” (i.e., in regions). Hence, it is quantitatively operationalized in a binary manner, i.e., regions with RPANs are given a score of 100, while those without are given a score of 0.

The indicators are summarized in Table 2.

### 2.3 Construction of the composite index

Principal Component Analysis (PCA) was conducted to determine the indicator groupings (referred to in this study as ‘domains’). High and moderate loadings (>0.50) were considered in assessing how the individual indicators are related to the principal components (22). Subsequently, data were normalized using Min-Max method, and transformed into positive values on a scale of 0 to 100 (with 100 being the highest score, and 0 being the lowest score). Equal weights were applied, scaled to unity sum, based on the premise that all indicators are equally important and interconnected in the food system. The aggregation method applied in the present study is the linear additive approach, i.e., domain scores were derived by the summation of weighted indicator scores.

### 2.4 Test of robustness

While there is no standard approach in data modeling for index development, the arbitrariness involved in each step of the development process puts into issue the robustness of the composite index. In the present study, uncertainty analysis and sensitivity test were conducted to provide transparency to the robustness of the data modeling approach employed. The uncertainty factors considered were the normalization methods, aggregation schemes, and inclusion/ exclusion of indicators. For sensitivity analysis, Kruskal-Wallis test was employed to determine the extent of difference in mean rankings in pairwise comparisons using different uncertainty factors.

### 2.5 Adaptation of the index to assess the status of food systems in the Philippine regions

To demonstrate the utility of the composite index, it was used to describe the status of the food systems among seventeen (17) regions of the Philippines.

## 3 Results

**Figure 2:**
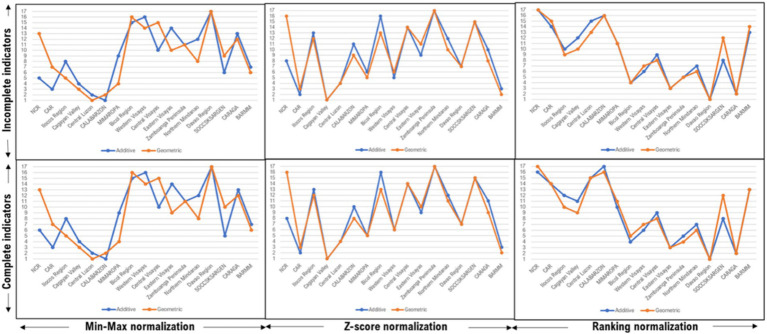
Uncertainty test results.

Based on the pattern and correlation matrices in the PCA outputs, the indicators were grouped into 4 domains (Table 3). The test of robustness indicated that there is a relative ‘shift’ in regional rankings evident when different normalization and methods are employed in each model. However, the application of different aggregation techniques and exclusion of an indicator did not cause a significant shift in the regional rankings (Figure 2). Results from the sensitivity analysis indicate that the in general, the choice of normalization method, aggregation scheme, and weightings significantly affects the rankings; however, the exclusion of an indicator generates rankings that are not significantly different.

**Table 3:** Summary of indicators.

Indicator	Definition	Variable(s)	Algorithm	Data source	Data access
Food supply diversity	Diversity of food commodities in the food supply in a given geographic area	Volume of crop production Volume of poultry and livestock production Volume of fisheries production	Shannon diversity (4)	Philippine Statistics Authority (PSA) Regional Agricultural Production Accounts 2019 (published 2021)	Publicly available downloadable report
Population share with adequate energy and nutrient intakes	Proportion of households in a given geographic area meeting the recommended intake levels for energy and (selected) nutrients	Proportion of households with energy intakes meeting 100% Recommended Energy Intake (REI) Proportion of households with intakes meeting the Estimated Average Requirement (EAR) for protein, iron, calcium, vitamin A, vitamin C, thiamin, riboflavin, and niacin	Average value of the variables	Department of Science and Technology-Food and Nutrition Research Institute (DOST-FNRI) Philippine Nutrition Facts and Figures Dietary Survey 2015 (published 2016)	Publicly available downloadable report
Food affordability	Ratio of the cost of nutritious diet to the minimum wage rate in a given geographic area	Estimated cost of nutritious diet by region	Cost of nutritious diet divided by the average minimum wage rate	World Food Programme (WFP) Fill the Nutrient Gap: Philippines Nutrition Situation 2018 (published 2019)	Summary report publicly available; requested access to full report
		Minimum wage rate by region		PSA Minimum wage rates by sector and by region 2018	Requested access
Food expenditure status	Share of household income spent on food	Percent to the total expenditure on food	Values adopted directly from data source	PSA Family Income and Expenditure Survey (FIES) 2019 (published 2020)	Publicly available downloadable report
Poverty incidence	Proportion of families/individuals with per capita income less than the per capita poverty threshold to the total number of families/individuals	Estimates of poverty incidence among families	Values adopted directly from data source and deducted from 100	PSA Official Poverty Statistics of the Philippines (published 2020)	Publicly available downloadable report
Water, sanitation and hygiene (WASH)	Percentage of families in a given geographic area with access to basic service level of drinking water, sanitation, and handwashing facilities	Percentage of families by service level of drinking water Percentage of families by service level of sanitation facilities Percentage of families by service level in which handwashing facilities were observed	Average value of the variables	PSA Annual Poverty Indicators Survey (APIS) 2019 (published 2020)	Publicly available downloadable report
Access to land	Proportion of fully owned holding/ farm parcels in a given geographic area	Number of fully owned holdings Total number of farm holdings or farm parcels	Number of fully owned holdings/farm parcels divided by the total number of farm holdings/farm parcels	PSA Census of Agriculture and Fisheries (CAF) 2012 (published 2017)	Publicly available downloadable report
Irrigation development	Rate of growth of service areas provided by irrigation system in a given geographic area	Percentage of irrigation development	Values adopted directly from data source	PSA Agricultural Indicators Survey (AIS) Government Support in the Agriculture Sector 2019 (published 2020)	Publicly available downloadable report
Support to technological transformation	Ratio of R&D expenditure to the Gross Domestic Product (GDP) of a given geographic area	Ratio of R&D intensity	Values adopted directly from data source	Department of Science and Technology (DOST) Compendium of S&T Statistics 2018 (published 2021)	Publicly available downloadable report
Road density	Ratio of the length of total road network (motorways, highways, main and secondary roads, etc.) to land area in a given geographical area	Road network	Values per city/municipality were grouped according to region and regional total scores were derived	Department of Trade and Industry (DTI) Cities and Municipalities Competitiveness Index (CMCI) 2019	Extracted from the portal
Technological capacity and readiness	Number of internet and telephone providers, and availability of public transport vehicles in a given geographic area	Information technology capacity	Values per city/municipality were grouped according to region and regional total scores were derived	DTI CMCI 2019	Extracted from the portal
Food security status	Proportion of food secure households in a given geographic area	Proportion of food secure households by region	Values adopted directly from data source	DOST-FNRI Philippine Nutrition Facts and Figures Food Security Survey 2015 (29)	Publicly available downloadable report
Plate waste	Percentage share in the amount of household plate waste of a given geographic area	Mean one-day household plate waste by food group and region	Values adopted directly from data source and deducted from 100	DOST-FNRI Philippine Nutrition Facts and Figures Dietary Survey 2015 (published 2016)	Publicly available downloadable report
Policy environment for resilience	Presence of emergency response/disaster risk reduction plans and budget allocation for such plans in a given geographic area	Presence of disaster risk reduction plan Budget for Disaster Risk Reduction and Management Program (DRRMP)	Average values for variables per city/municipality were grouped according to region and regional total scores were derived	DTI CMCI 2019	Extracted from the portal
Policy environment for nutrition	Presence of nutrition policies/action plans in a given geographic area	Presence of nutrition action plan	Binary scoring (100 = with Regional Plan of Action for Nutrition (RPAN); 0 = without RPAN)	(21)	Publicly available downloadable documents

Regional average aggregated scores for the four (4) domains and the corresponding regional rankings (Table **4**) show the overall status of the food systems in the regions, with higher scores indicating a ‘better’ status and lower scores implying an ‘inferior’ status. Central Luzon, CALABARZON, and CAR have the highest total scores implying that the food system status in these regions is better among all the regions in the country. Meanwhile, Bicol Region, Western Visayas, and Davao Region obtained the lowest scores.

**Table 4:** Average regional composite index scores and corresponding ranks.

Region	Aggregated Score	Rank
National Capital Region (NCR)	50.47	6
Cordillera Autonomous Region (CAR)	55.85	3
Ilocos Region (Region I)	48.74	8
Cagayan Valley (Region II)	54.27	4
Central Luzon (Region III)	64.34	2
CALABARZON (Region IV-A)	64.70	1
MIMAROPA (Region IV-B)	46.90	9
Bicol Region (Region V)	38.07	15
Western Visayas (Region VI)	35.41	16
Central Visayas (Region VII)	46.24	10
Eastern Visayas (Region VIII)	39.13	14
Zamboanga Peninsula (Region IX)	43.30	11
Northern Mindanao (Region X)	43.30	12
Davao Region (Region XI)	26.09	17
SOCCSKSARGEN (Region XII)	50.63	5
Caraga (Region XIII)	40.45	13
Bangsamoro Autonomous Region in Muslim Mindanao (BARMM)	48.88	7

**Figure 3:**
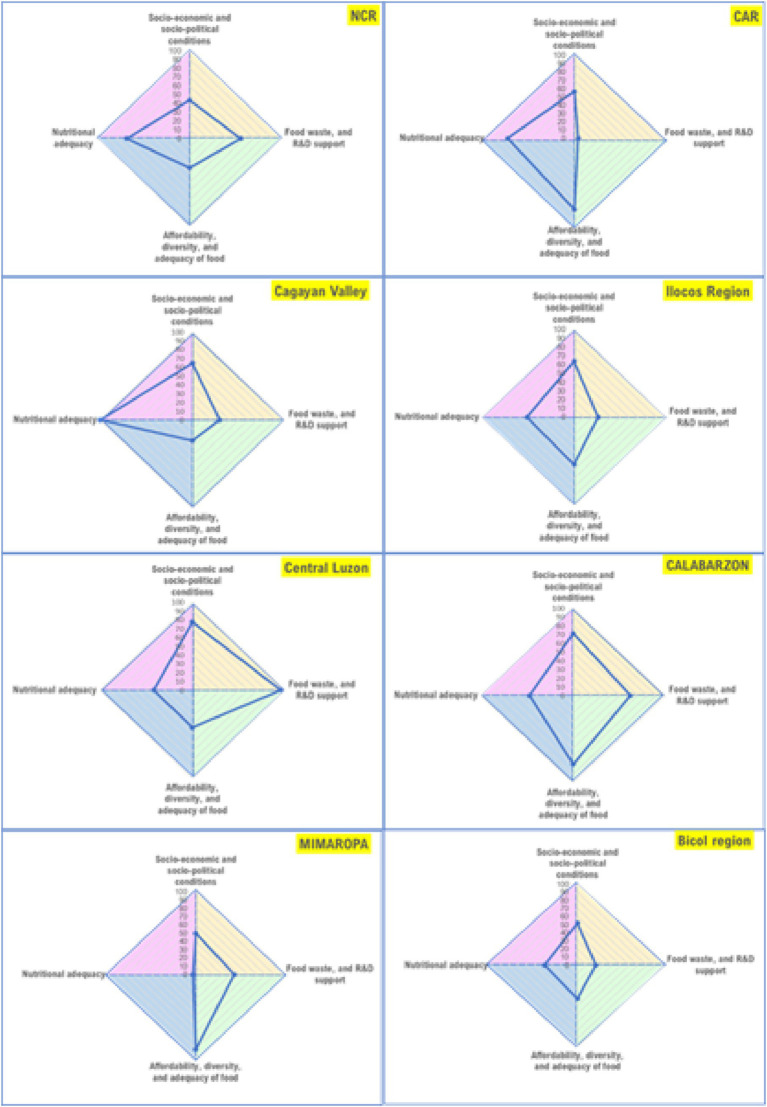
Region-level food system status in Luzon (areas of the polygon represent the status of the food system per domain, with higher scores indicating better status).

**Figure 4:**
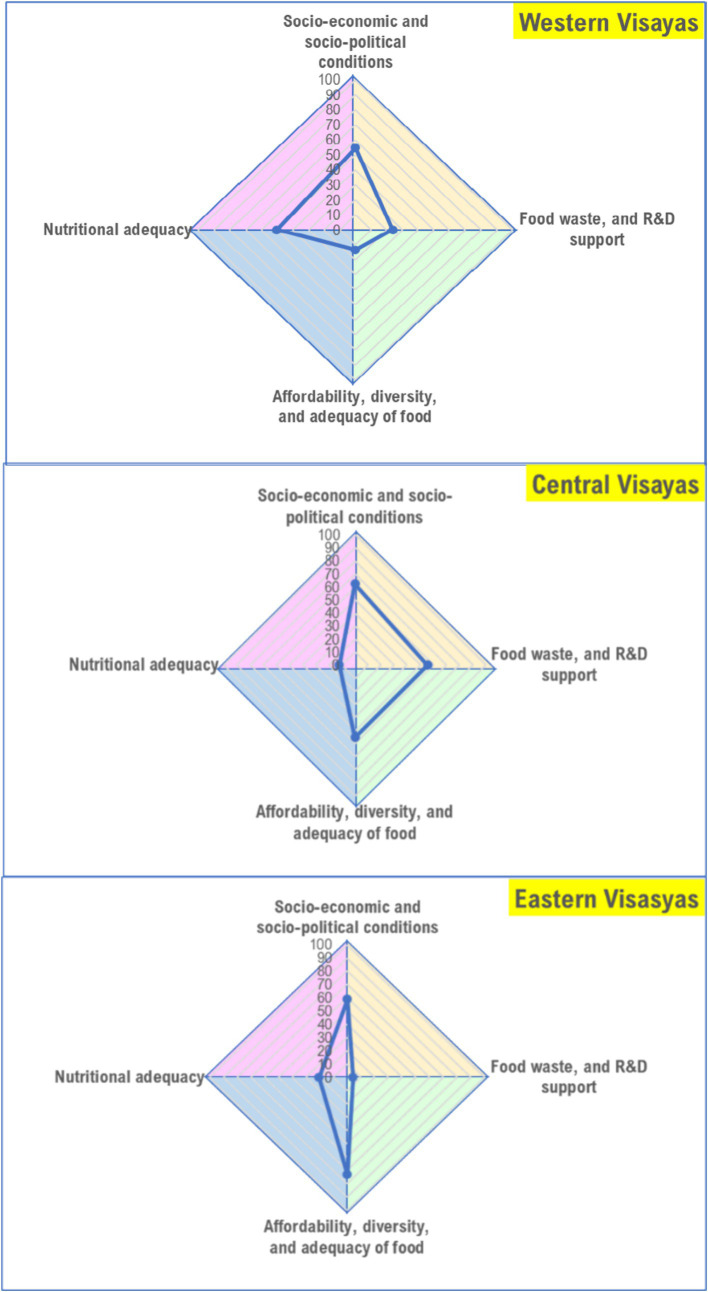
Region-level food system status in Visayas (areas of the polygon represent the status of the food system per domain, with higher scores indicating better status).

**Figure 5:**
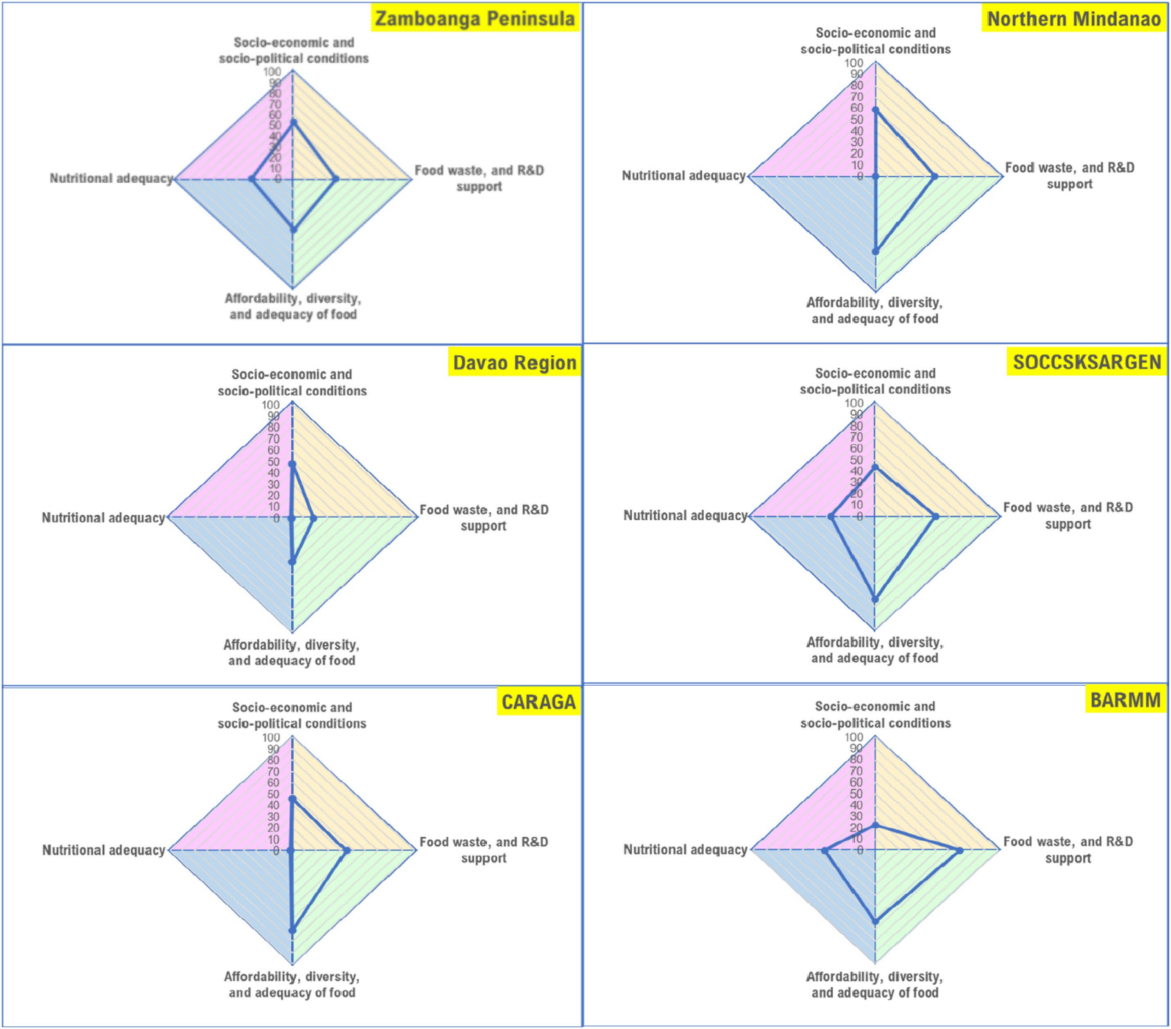
Region-level food system status in Mindanao (areas of the polygon represent the status of the food system per domain, with higher scores indicating better status).

Regions within each main island (Luzon, Visayas, and Mindanao) scored differently across domains (Figure 3). In Luzon, CALABARZON and Central Luzon have higher scores in terms of socio-economic and political conditions as compared with the other regions, but the score on nutritional adequacy is both low in these regions; while Cagayan Valley, CAR, and NCR have higher scores on this domain. Scores on diversity, affordability, and adequacy of food in CAR, MIMAROPA, and CALABARZON is relatively higher than the other regions; NCR, Cagayan Valley, and Bicol region have low scores for this domain. MIMAROPA obtained the lowest score the domain for nutritional adequacy; Bicol region and Central Luzon also obtained low scores; while Cagayan Valley, CAR, and NCR have relatively higher scores. Scores for food waste and R&D support is low in CAR, Ilocos Region, Cagayan Valley, and Bicol Region; but Central Luzon, CALABARZON, and NCR have better scores for this domain. Similarly, regions in Visayas scored differently across domains (Figure 4). The score for food affordability, diversity, and adequacy is lowest in Western Visayas but the region’s score for nutritional adequacy is higher relative to the two (2) other regions. Food waste and R&D support score is lowest in Eastern Visayas, but its score for the domain socio-economic and socio-political conditions is almost comparable with the other regions in the island. In Mindanao, nutritional adequacy domain scores are generally low across regions, particularly in Northern Mindanao, Davao Region, and Caraga (Figure 5). Food waste and R&D support score is lowest in the Davao Region, and highest in BARMM, but the latter obtained the lowest score on the domain socio-economic and socio-political conditions.

## 4 Discussion

Several metrics for food systems assessment exist (2–4, 7, 23) in the international literature. Although these indicators are inarguably valuable, their utility and relevance to the Philippine context may be limited primarily of three reasons: (i) the formulation of these indicators are globally contextualized without considering country differences in institutional structures, ecological conditions, and resources; (ii) the data required to operationalize these indicators are normally obtained from global databases and as such, (iii) food system assessments are limited at the country level. The development of contextually fit composite index for food system assessment in the Philippines is an attempt to address these gaps. To the best of our knowledge, this is the first endeavor in the country that makes use of locally available pertinent data for the development of quantifiable set of indicators intended for food system assessment at the sub-national scales.

We obtained data mostly from government databases in the country, thereby increasing the significance of conducting localized assessment for food system transformations in addition to improving the utility value of existing data sources. For example, the PSA data on minimum daily wage rates is typically used to assess sectoral and regional compliance to work orders issued by the government. In this study however, this set of information is given additional application by using it to determine the sufficiency of the prevailing minimum daily wages to afford a nutritious diet in the formulation of the indicator Food Affordability. The premise by which this indicator is formulated is pertinent to the country’s current economic and food security challenges, and its inclusion in food system assessment could serve as a basis to identify alternatives to improve affordability of nutritious foods. Similarly, the indicator Food Supply Diversity utilizes the PSA’s agricultural production data to assess the capacity of local food systems to support the delivery of a diverse range of food items for better diet quality. Again, the formulation of this indicator adds utility value to the locally available agricultural statistics and provides the opportunity for stakeholders to evaluate the effectiveness of current programs and policies on agricultural diversification in the country.

The set of indicators in the present study makes use of data that are available at the national and sub-national (region) levels. Thus, following the notion that the LGUs are in better position to identify problems and craft appropriate solutions to address them, and working on the premise that different agroecological conditions consequently affect food systems differently, the metrics developed in the present study can be utilized by government leaders, policy makers, and other stakeholders to inform decisions in resource allocation, and in the development of measurable and actionable policies and/ or programs that would be more responsive to the needs of their respective communities.

The findings in this study indicate that overall, food system concerns and priorities vary, and thus approaches to address them need to be context-specific. The nature and extent of the problems confronting the geographical regions in the country are not the same and as such, program and policy priorities differ. Case in point, CAR, MIMAROPA, and CALABARZON have low scores on the nutritional adequacy domain while Cagayan Valley scored better in this domain although, on the other hand, it scored low on the food diversity, affordability, and adequacy domain. This implies that strategies to increase the proportion of households with adequate energy and nutrient intakes should be prioritized in CAR, MIMAROPA, and CALABARZON while mechanisms for improving food production and supply diversity and affordability of food are the main considerations for Cagayan Valley. In Western Visayas, the food system is challenged in terms of being able to provide diverse, affordable, and adequate diets. The score for the domain food waste and R&D support (measured in terms of regional GDP allocation for R&D) is also low in the region, and thus, food system priority areas should consider these domains in this region. Central Visayas, on the other hand, has a better score on food waste and R&D support, but needs to consider programs that would improve the nutritional adequacy of diets for the population; while the food system of Eastern Visayas is challenged in both domains. In Mindanao, regional domain scores also vary although in general, the scores for the nutritional adequacy domain are low across regions.

The preceding instances highlight the need for region-specific approaches to improve the delivery of outcomes through a food system approach. The sub-national food system assessment provided in this study provides the local decision-makers with relevant information on the status of their respective food systems, making them better equipped to understand the problems confronting their food system, define clearer goals, prioritize resources, and establish realistic timelines. The concept of “local universality” describes the process where general rules, products, or guidelines are shaped and tailored to fit into local contexts and enacted within practices (24) – a phenomenon that can be described as “dispersed governance.” Even when governance is centralized, policy and program implementation are still highly dependent on local context, which means that an intervention that has been reportedly successful in one location does not necessarily mean that it would deliver the same results in another location (25, 26). This is particularly relevant in a country like the Philippines where authority, responsibility, and resource allocation and management are decentralized to the local government, and with the strengthened fiscal decentralization in the Mandanas-Garcia Supreme Court ruling. Considering that local governments are empowered to identify and decide on their development programs and interventions, providing region-specific information on food system status allows local authorities to determine their respective priority areas and design initiatives that are more contextually fit, instead of simply following blanket national strategies. This perspective is aligned with the observations in a recently published report where it was highlighted that although most food system issues are shared, priorities differ driven by the variation in market dynamics, governance, policies and regulations, demographic landscape, environmental structure, social and cultural factors, and technological and financial access (27).

The development of the composite index was conducted with academic rigor, and the food system scores derived in this study provide a quantitative assessment of the status of the local food systems at the region-level. The authors recognize the importance of the interactions across food system elements, actors, and drivers which are not captured in the present study. In Mindanao for instance, scores on the nutritional adequacy domain are low across regions which brings the following interesting questions: (i) “which among the other domains considerably affects nutritional adequacy in each region: is it socio-economic and socio-political conditions, food waste and R&D support, or food affordability, diversity, and adequacy?,” and (ii) “will there be improvements in nutritional adequacy if the score in another domain is increased?”

The conduct of further analyses to deepen the understanding of the interrelationships of the elements in the local food systems would be helpful in the formulation of specific integrated multisectoral strategies for the improvement of food system status. This approach was adopted in the Multidimensional Poverty Index (MPI) report where different combinations of the indicators across the three (3) dimensions of poverty measured (i.e., health, education, and standards of living) were analyzed to assess the overlapping deprivations (pertained to as deprivation profiles) across different countries to deepen understanding of the texture and variations of poverty to guide policymakers on specific interventions that would be more meaningful to the population (28). While this study did not include similar analyses to provide sufficient information to support the development of intersectoral strategies in the Philippine food system, it can be used as a starting point for the conduct of relevant analyses to better understand pathways for the regions to address specific food system concerns. Currently, policies and programs in the country tend to be in distinct siloes despite the growing academic promotion of intersectoral partnerships. The imperative of the developed composite index therefore, is for policy design to be considered as an integrative process for putting forward diet quality and nutrition as a common ground.

We further acknowledge a number of methodological considerations in the present study. For one, we used secondary data in developing the algorithms for the indicators and these data have inherent uncertainties and limitations. Further, we recognize that indicators to assess the environmental elements, resilience, and food safety dimensions of the local food systems are not included in the current set of indicators we developed owing to the limited availability and completeness of pertinent data at this time. We also acknowledge that the set of indicators we developed need to be further improved by testing its reliability and applicability before it can be considered a good assessment tool. The conduct of field tests at different geographic scales, (i.e., national, regional, provincial, and/or municipal levels) and at different time frames to examine these characteristics is recommended.

## 5 Conclusion

A growing body of evidence recognize the importance of context-specific food system assessment to improve relevance of findings to facilitate food system transformation endeavors. The most important contribution of this study is the generation of a locally developed assessment tool that demonstrated usefulness in providing information on the status of local food systems in the Philippines by quantitatively characterizing the status of the food systems in the seventeen (17) regions of the country. Results highlighted differences food system concerns and priority areas, providing implications to guide the development of context-specific programs and policies.

## Data Availability

The original contributions presented in the study are included in the article/supplementary material, further inquiries can be directed to the corresponding author/s.

## Footnotes

^1^ https://cmci.dti.gov.ph/data-portal.php
